# Supplementary material for: Network-based prediction of protein interactions
Source: Nat Commun. 2019 Mar 18;10:1240. doi: 10.1038/s41467-019-09177-y (PMC6423278; doi:10.1038/s41467-019-09177-y)
Supplement: Supplementary file 1 — Supplementary Information [file 41467_2019_9177_MOESM1_ESM.pdf]

Network-based Prediction of Protein Interactions

Kovács et al., *Nature Communications* 2019.

SUPPLEMENTARY INFORMATION

Supplementary Information includes:

Supplementary Notes **1** to **2**.

Supplementary Tables **1** to **3**.

Supplementary Figures **1** to **10**.

Supplementary References.

Supplementary Notes

Supplementary Note 1

Experimental Details. Yeast strains expressing the proteins to be tested were each picked as bait (DB fusions, in yeast strain Y8930C) and prey (AD fusions, in yeast strain Y8800C) such that every interaction was tested in both orientations (DB-X with AD-Y and DB-Y with AD-X). In cases where a protein is known to auto-activate the expression of the reporter gene when fused to the DB domain, the corresponding interaction was only tested with that protein fused to the AD domain, while the other orientation is classified as undetermined. To perform the pairwise test, strains expressing the bait and prey protein fusions were inoculated in selective medium (200μl Synthetic Complete [SC] medium lacking leucine (-Leu) for DB-fusion proteins, and 200μl SC lacking tryptophan (-Trp) for AD-fusion proteins) using the liquid handling robot. After 48h incubation at 30$°C$ on a vibrating shelf, the appropriate bait and prey strains were mated by transferring 5μl of each into 120μl rich (YPD) media. The next day, 10μl of the mated yeast was transferred into 120μl SC-Leu-Trp media to select for diploids. After 24h of incubation at 30$°C$, the yeast were spotted on solid media (SC-Leu-Trp-His + 1 mM 3AT, test plate) to select for cells expressing the GAL1::HIS3 reporter gene. Cells were also spotted on SC-Leu-His + 1 mM 3AT + 1 mg/L cyclohexamide (CHX plate) (Sigma-Aldrich, # C7698-5G) to select for de novo auto-activation, i.e. activation of the reporter gene by the DB-protein fusion alone as result of spontaneous mutations that occurred after inoculation [28]. After incubation for three days at 30$°C$ and an additional two days at room temperature, the plates were scored by comparing for every protein pair the strength of growth of the yeast colonies on the test plate with the strength of growth on the CHX plate. A protein pair was scored as positive only when significantly more growth was observed on the test plate compared to the CHX plate. In the case of too strong growth on CHX plate, a pair was scored as auto-activator (classified as undetermined). If there was no growth on test and CHX plate, the pair was scored as negative. If there was no yeast spotted on the plate, the pair was scored as invalid (undetermined).

To confirm the identity of the interacting bait and prey fusion proteins, colonies of all pairs scored as positive were picked into SC-Leu-Trp selection media and incubated overnight at 30$°C$ on a vibrating shelf. These cultures were lysed by transferring 5μl of the yeast culture into 25μl of lysis buffer (81% of 0.2M dibasic sodium phosphate (Fisher Scientific, #S369500 and 19% 0.2 monobasic sodium phosphate (Fisher Scientific, #S373500), final pH of 7.5 in the presence of Zymolase (US Biological Life Science, #Z1000)) followed by incubation for 3h at 37$°C$ in a 96-well PCR plate. The Zymolase was inactivated by a brief heat shock (5 min at 65$°C$). The lysate was diluted by adding 80μl sterile water and then was centrifuged for 10 minutes at 1500 rpm. A PCR was performed using Platinum Taq (#11304102, Life Technologies) and universal forward primers for AD (5'-CGCGTTTGGAATCACTACAGGG-3') and DB (5'-GGCTTCAGTGGAGACTGATATGCCTC-3') as well as a common reverse primer (5'-GGAGACTTGACCAAACCTCTGGCG-3'). The PCR products were confirmed by gel electrophoresis and sent for Sanger sequencing (Genewiz). The traces were aligned to the expected ORF sequences. A protein pair previously scored as positive remained positive (sequence confirmed) only if there was a well that unambiguously identified the corresponding pair of ORFs, otherwise it is classified as undetermined. Overall, the number of negative pairs is expected to be overestimated due to sampling sensitivity and the fact that there was no sequence confirmation step for negative pairs, some of which might not be unambigously identifiable, masking an undetermined pair.

As a result, each pair in each orientation is categorized to be either positive, negative or undetermined. To summarize the information obtained for both orientations, the pair is "positive" if any of the orientations are positive and "negative" if all orientations scored negative. Note, that for self-interaction we have a

single orientation only. The overall recovery rate is measured as the fraction of positives over positives and negatives. Besides the result shown in Fig. 3, we obtained the following recovery rates: PRS: 25 ± 9% (7 out of 28), Lit-BM-13: 15 ± 7% (5 out of 33) RRS: 0% (0 out of 68), indicating an experiment, efficiently differentiating between random and interacting pairs of proteins. Note that although HI-tested is substantially smaller than HI-II-14, it performs at least as well in the high-throughput (HT) test as HI-II-14 (Supplementary Fig. 6).

Supplementary Note 2

Quantitative measures to evaluate the predictive power. There are several popular measures to quantify predictive power of classification methods. In our quality assessments we relied on the following three measures.

1) Area Under the receiver operating characteristic Curve (AUC) is calculated as [33]

$$AUC = \frac{n' + 0.5n''}{n} , \tag{1}$$

where by randomly selecting n pairs of a positive and negative link, we obtain larger score n' times and an equal score n'' times for the positive link. The AUC value is 0.5 for no predicting power and a higher value (up to 1) is assumed to indicate a better performance.

2) The Precision is defined as [34]

$$P = \frac{L_p}{L} , \tag{2}$$

where L_p is the number of positive links in the top L number of predictions. The precision is between 0 and 1 and higher precision value means higher accuracy. 3) The normalized Discounted Cumulative Gain (nDCG) is proven to be able to select the better ranking between any two, substantially different rankings [35]. For binary classification the nDCG is given by

$$nDCG = \frac{\sum_{i \in P} \frac{1}{\log_2(i+1)}}{\sum_{i=1}^{|P|} \frac{1}{\log_2(i+1)}} , \tag{3}$$

where the summation in the nominator runs over all positive instances, while the summation in the denominator quantifies the ideal case, where the positive instances are the top ranked predictions. In practice, to deal with extreme degeneracies, for the candidates with a zero score, we assigned the last possible rank to each of them. The obtained performance values are summarized in Supplementary Table 3 for all studied networks.

Supplementary Tables

Supplementary Table 1: **Link prediction methods, implemented and tested.** Γ_i stands for the set of neighbors for node i and $k_i = |\Gamma_i|$ stands for the degree, while $\gamma_i = \{i\} \cup \Gamma_i$. κ_z is the subset of neighbors of z that are also neighbors of i and j. e_i is the external degree if i, considering nodes only that are not neighbors of j. In YZ, d_{ij} is the distance of nodes i and j, while for LP the value of $\beta = 0.01$ is used. D stands for the diagonal degree matrix with entries $D_{ii} = k_i$. In HG N is the number of nodes in the network. k_{av} is the average degree and $\lambda_{ij} = \max(0, k_{av} - (|\gamma_i - \gamma_j| + |\gamma_i \cap \gamma_j|))$. Our list is not intended to be complete, showing only a sample of the simple, popular methods developed during in the last century. There are further network-based link prediction algorithms of higher computational complexity, such as Ref. [4], mostly dominated by TCP as the leading order term, such as the Katz index, or various random walk based techniques, such as the average commute time, PageRank or superposed random walk measures [5], as well as methods relying on spatial proximity [40, 41]. Yet, TCP or similarity-based link prediction tools are generally expected to be inefficient for PPI datasets. While $\ell = 3$ paths have been used before for link prediction in bipartite datasets [19, 20], in this paper we show that L3 is an efficient framework for PPI networks, even though PPI networks are far from being bipartite (Supplementary Table 2).

Method	Reference	Formula	Length
$\ell = 2$, CN (A^2, Common Neighbors)	[7]	$CN_{ij} = \|\Gamma_i \cap \Gamma_j\| = [A^2]_{ij}$	$\ell = 2$
$\ell = 3$ (A^3)		$A3_{ij} = [A^3]_{ij}$	$\ell = 3$
$\ell = 4$ (A^4)		$A4_{ij} = [A^4]_{ij}$	$\ell = 4$
JC (Jaccard index)	[22]	$JC_{ij} = \frac{CN_{ij}}{\|\Gamma_i \cup \Gamma_j\|}$	$\ell = 2$
HDI (Hub Depressed)	[6]	$HDI_{ij} = \frac{CN_{ij}}{\min(k_i, k_j)}$	$\ell = 2$
HPI (Hub Promoted)	[6]	$HPI_{ij} = \frac{CN_{ij}}{\max(k_i, k_j)}$	$\ell = 2$
SAL (Salton)	[6]	$SAL_{ij} = \frac{CN_{ij}}{\sqrt{k_i \times k_j}}$	$\ell = 2$
SEN (Sørensen)	[6]	$SEN_{ij} = \frac{2CN_{ij}}{k_i + k_j}$	$\ell = 2$
LHN (Leicht-Holme-Newman)	[6]	$LHN_{ij} = \frac{CN_{ij}}{k_i \times k_j}$	$\ell = 2$
AA (Adamic-Adar)	[31]	$AA_{ij} = \sum_{z \in \Gamma_i \cap \Gamma_j} \frac{1}{\log k_z}$	$\ell = 2$
RA (Resource Allocation)	[32]	$RA_{ij} = \sum_{z \in \Gamma_i \cap \Gamma_j} \frac{1}{k_z}$	$\ell = 2$
LCL (Local Community Links)	[9]	$LCL_{ij} = \sum_{z \in \Gamma_i \cap \Gamma_j} \frac{\|\kappa_z\|}{2}$	$\ell = 2, 3$
CAR	[9]	$CAR_{ij} = CN_{ij} \times LCL_{ij}$	$\ell = 2$
CPA	[9]	$CPA_{ij} = e_i \times e_j + (e_i + e_j) \times CAR_{ij} + CAR_{ij}^2$	$\ell = 2$, other
CAA	[9]	$CAA_{ij} = \sum_{z \in \Gamma_i \cap \Gamma_j} \frac{\|\kappa_z\|}{\log_2 \|\Gamma_z\|}$	$\ell = 2, 3$
CRA	[9]	$CRA_{ij} = \sum_{z \in \Gamma_i \cap \Gamma_j} \frac{\|\kappa_z\|}{\|\Gamma_z\|}$	$\ell = 2, 3$
CJC	[9]	$CJC_{ij} = \frac{CAR_{ij}}{\|\Gamma_i \cup \Gamma_j\|}$	$\ell = 2$
HG (Hypergeometric)	[39]	$HG_{ij} = -\log \sum_{n=CN_{ij}}^{\min(k_i, k_j)} \frac{\binom{k_i}{n}\binom{N-k_i}{k_j-n}}{\binom{N}{k_j}}$	$\ell = 2$
PA (Preferential Attachment)	[8]	$PA_{ij} = k_i \times k_j$	other
LP (Local Path)	[26]	$LP_{ij} = [A^2 + \beta A^3]_{ij}$	$\ell = 2, 3$
YZ (Yang-Zhang)	[25]	$YZ_{ij} = \frac{CN_{ij} + 1}{d_{ij}}$	$\ell = 2$, other
(-1) CDD (Czekanowski-Dice Dissimilarity)	[12]	$CDD_{ij} = \frac{\|\gamma_i \cup \gamma_j\| - \|\gamma_i \cap \gamma_j\|}{\|\gamma_i \cup \gamma_j\| + \|\gamma_i \cap \gamma_j\|}$	$\ell = 2$
(-1) ACDD (Adjusted CDD)	[13]	$ACDD_{ij} = \frac{\|\gamma_i \cup \gamma_j\| - \|\gamma_i \cap \gamma_j\| + \lambda_{ij} + \lambda_{ji}}{\|\gamma_i \cup \gamma_j\| + \|\gamma_i \cap \gamma_j\|}$	$\ell = 2$
FSW (Functional Similarity Weight)	[10]	$FSW_{ij} = \frac{4\|\gamma_i \cap \gamma_j\|^2}{(\|\gamma_i - \gamma_j\| + 2\|\gamma_i \cap \gamma_j\| + \lambda_{ij})(\|\gamma_j - \gamma_i\| + 2\|\gamma_i \cap \gamma_j\| + \lambda_{ji})}$	$\ell = 2$

Supplementary Table 2: **Analyzed networks and their main characteristics** N: number of nodes, E: number number of interactions, k_{av}: average degree, ρ: network density, d_{max}: maximum graph distance, d_{av}: average graph distance, C: clustering coefficient, SIPs: number of self-interacting proteins. We test our results on several interactomes covering seven species. We start with systematic networks generated by a binary pipeline, such as the human (HI-II-14, [1]), yeast (Y2H-Union [23]) and arabidopsis (AI [24]) interactomes. We continue with literature-curated PPI networks of direct physical interactions, such as Lit-BM-13 [1], or the Biogrid datasets [21]. From BioGRID, we only considered "direct interactions" and proteins assigned to the correct "taxid", specific to the studied species. We also considered co-complex proteomics datasets, such as the Bioplex [3] and Hein et al. [14] AP-MS datasets. For completeness we also included the Interactome3D [2] dataset, summarizing currently available interactions with structural evidence.

Species	Dataset	Reference	N	E	k_{av}	ρ	d_{max}	d_{av}	C	SIPs
H. sapiens	HI-II-14	[1]	4298	13868	6.212	$1.45E-3$	11	4.067	0.052	518
H. sapiens	HI-tested	here	3727	9433	4.823	$1.29E-3$	11	4.304	0.025	445
H. sapiens	HI-III	[18]	5604	23322	8.208	$1.47E-3$	10	3.851	0.068	322
H. sapiens	Lit-BM-13	[1]	5545	11045	3.663	$6.61E-4$	15	5.306	0.063	890
H. sapiens	Human-BioGRID	[21]	12595	66542	10.313	$8.19E-4$	10	3.651	0.073	1596
H. sapiens	Bioplex	[3]	10961	56553	10.319	$9.42E-4$	11	4.279	0.095	–
H. sapiens	Hein et al.	[14]	5457	28780	10.135	$1.86E-3$	9	3.775	0.158	1127
H. sapiens	Interactome3D	[2]	5291	8880	2.281	$4.31E-4$	25	7.676	0.097	2846
S. cerevisiae	Yeast	[23]	2018	2930	2.681	$1.33E-3$	14	5.611	0.046	225
S. cerevisiae	Yeast-BioGRID	[21]	4885	28270	11.161	$2.29E-3$	10	3.603	0.120	1009
A. thaliana	Arabidopsis	[24]	4865	11374	4.492	$9.24E-4$	14	5.180	0.098	446
A. thaliana	Arabidopsis-BioGRID	[21]	7620	29241	7.491	$9.83E-4$	15	4.807	0.099	701
C. elegans	Worm-BioGRID	[21]	3153	5540	3.420	$1.09E-3$	13	4.780	0.027	148
D. melanogaster	Fly-BioGRID	[21]	7283	23952	6.522	$8.96E-4$	12	4.329	0.013	201
S. pombe	S. Pombe-BioGRID	[21]	1929	3700	3.397	$1.76E-3$	14	4.671	0.064	424
M. musculus	Mouse-BioGRID	[21]	2092	3054	2.788	$1.33E-3$	17	5.999	0.048	138

Supplementary Table 3: **Computational cross-validation.** P stands for precision in Eq. (2), ie. the validation rate, evaluated at the top 2000 predictions. nDCG is the normalized Discounted Cumulative Gain in Eq. (3), a frequently used measure of quantifying ranking quality. AUC is the Area Under the receiver operating characteristic Curve in Eq. (1), i.e., the probability that a randomly chosen validated link has a lower rank than a not validated link. The number in the parentheses indicates the standard deviation of the measures over 10 realizations in terms of the last provided digit (only values greater than zero are shown). "B." abbreviates BioGRID. The highest scores are indicated in bold for each dataset and each measure. While methods relying on paths of specific lengths are constrained in their range, PA is able to predict all protein pairs, typically leading to a high global AUC score. Nevertheless, measures focusing on the top, high-quality predictions, such as the Precision and nDCG values, are able to indicate the improved performance of L3.

Dataset	L3			A3			CRA			CN			PA		
	P	nDCG	AUC	P	nDCG	AUC	P	nDCG	AUC	P	nDCG	AUC	P	nDCG	AUC
HI-II-14	**0.335(7)**	**0.697(2)**	0.786(4)	0.322(6)	0.687(3)	0.785(4)	0.104(7)	0.510(2)	0.517(2)	0.092(8)	0.541(3)	0.587(5)	0.093(4)	0.647(1)	**0.806(4)**
HI-tested	**0.168(8)**	**0.619(2)**	0.717(3)	0.157(9)	0.611(3)	0.716(3)	0.004	0.461(2)	0.501	0.022(2)	0.483(1)	0.536(3)	0.058(3)	0.608(2)	**0.753(3)**
HI-III	**0.446(7)**	**0.744(2)**	**0.849(2)**	0.431(8)	0.730(1)	0.845(2)	0.194(5)	0.541(2)	0.530(2)	0.134(7)	0.582(2)	0.627(4)	0.113(4)	0.680(2)	0.848(2)
Lit-BM-13	**0.190(8)**	**0.567(2)**	0.626(3)	0.15(1)	0.555(3)	0.626(3)	0.013	0.455(2)	0.503	0.062(4)	0.492(2)	0.553(2)	0.038(2)	0.540(1)	**0.680(4)**
Human-B.	**0.40(1)**	**0.708(1)**	0.827(2)	0.38(1)	0.690(1)	0.822(1)	0.213(7)	0.532(1)	0.514(1)	0.159(8)	0.586	0.619(1)	0.097(5)	0.659	**0.859(1)**
Bioplex	**0.54(2)**	**0.721(1)**	0.786(1)	0.38(2)	0.703(2)	0.786(1)	0.321(8)	0.545(1)	0.522(1)	0.230(4)	0.587(1)	0.596(1)	0.028(4)	0.606	**0.807(1)**
Hein et al.	**0.606(8)**	**0.740(1)**	0.792(3)	0.54(1)	0.716(2)	0.788(2)	0.327(7)	0.585(3)	0.559(3)	0.28(1)	0.631(2)	0.650(3)	0.087(3)	0.646(1)	**0.842(2)**
Interactome3D	**0.228(5)**	**0.603(4)**	0.665(5)	0.176(7)	0.587(4)	0.665(5)	0.013	0.445(3)	0.505(1)	0.073(5)	0.485(2)	0.575(3)	0.024(3)	0.503(2)	**0.671(5)**
Yeast	**0.073(4)**	**0.515(6)**	0.582(4)	0.063(4)	0.505(8)	0.582(4)	0.001	0.415(5)	0.500	0.012(2)	0.431(2)	0.524(2)	0.040(2)	0.507(3)	**0.595(6)**
Yeast-B.	**0.410(8)**	**0.745(1)**	0.845(2)	0.37(1)	0.726(1)	0.840(2)	0.269(9)	0.562(1)	0.533(1)	0.205(9)	0.624(1)	0.664(3)	0.060(3)	0.658(1)	**0.847(2)**
Arabidopsis	**0.43(1)**	**0.690(2)**	**0.731(3)**	0.367(8)	0.673(2)	0.731(3)	0.051	0.481(2)	0.509(1)	0.054(3)	0.510(2)	0.575(4)	0.012(2)	0.568(1)	0.728(4)
Arabidopsis-B.	**0.661(8)**	**0.778(1)**	**0.839(2)**	0.650(8)	0.757(1)	0.838(2)	0.274(5)	0.530(1)	0.523(1)	0.048(8)	0.555(1)	0.617(2)	0.096(7)	0.670(1)	0.827(3)
Worm-B.	**0.091(3)**	0.529(2)	0.605(3)	0.076(4)	0.522(3)	0.605(3)	0.003	0.439(2)	0.501(1)	0.019(3)	0.459(2)	0.528(2)	0.044(3)	**0.544(2)**	**0.657(5)**
Fly-B.	**0.283(5)**	**0.606(1)**	0.668(2)	0.256(7)	0.602(1)	0.667(2)	0.009	0.483(1)	0.501	0.030(2)	0.501	0.528(1)	0.037(4)	0.598	**0.783(2)**
S. Pombe-B.	**0.141(5)**	**0.604(4)**	**0.663(4)**	0.119(6)	0.589(4)	0.662(4)	0.003	0.443(5)	0.502(1)	0.026(3)	0.469(3)	0.552(4)	0.074(4)	0.517(5)	0.582(4)
Mouse-B.	**0.074(4)**	**0.517(5)**	0.582(4)	0.065(6)	0.507(5)	0.582(4)	0.001	0.418(3)	0.501	0.019(2)	0.440(3)	0.527(2)	0.020(3)	0.490(3)	**0.595(6)**

Supplementary Figures

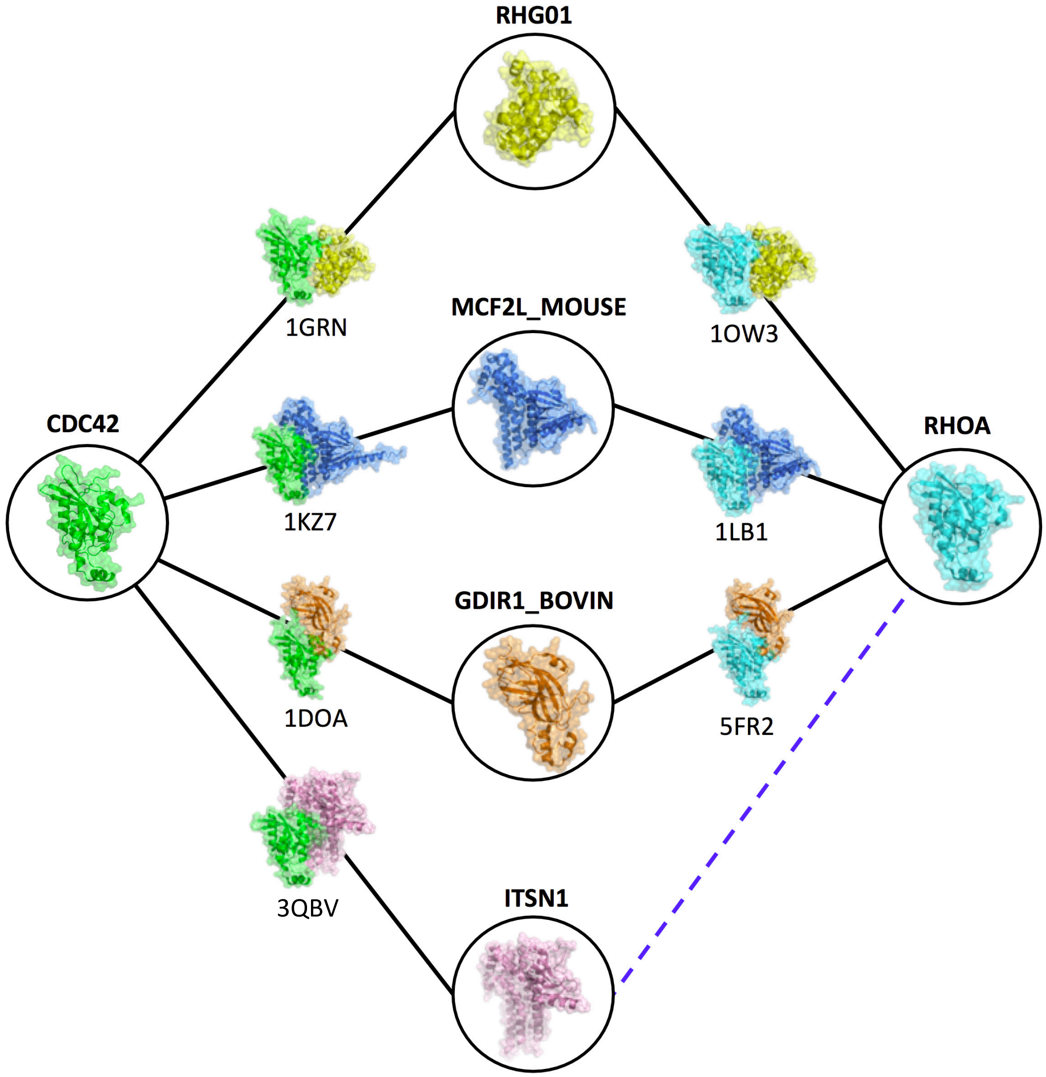

Supplementary Figure 1: **3D Structural illustration of the L3 principle.** To illustrate our link prediction pirnicple with existing 3D structural data, we selected two human proteins from PDB [27], CDC42 and RHOA, interacting with some of their partners through the same shared interface. While these two proteins are not known to interact with each other, we expect them to share some additional interacting partners, interacting with the same shared interface. From a network perspective, the structurally inferred (blue) interaction connects nodes that are linked by a larger number of paths of length $\ell = 3$. Besides ITSN1, CDC42 has two further interacting partners through the same interface, FMNL1 and FMNL2, potentially interacting with RHOA as well. Similarly, RHOA has also some interacting partners thorugh the same interface, potentially shared by CDC42: AKP13, ANLN, ARHG8, ARHGC, ARHGP and RHG20. This detailed example illustrates that even in the lack of any structural information, on the contrary of TCP-based methods, L3 might be able to infer missing interactions from the network topology.

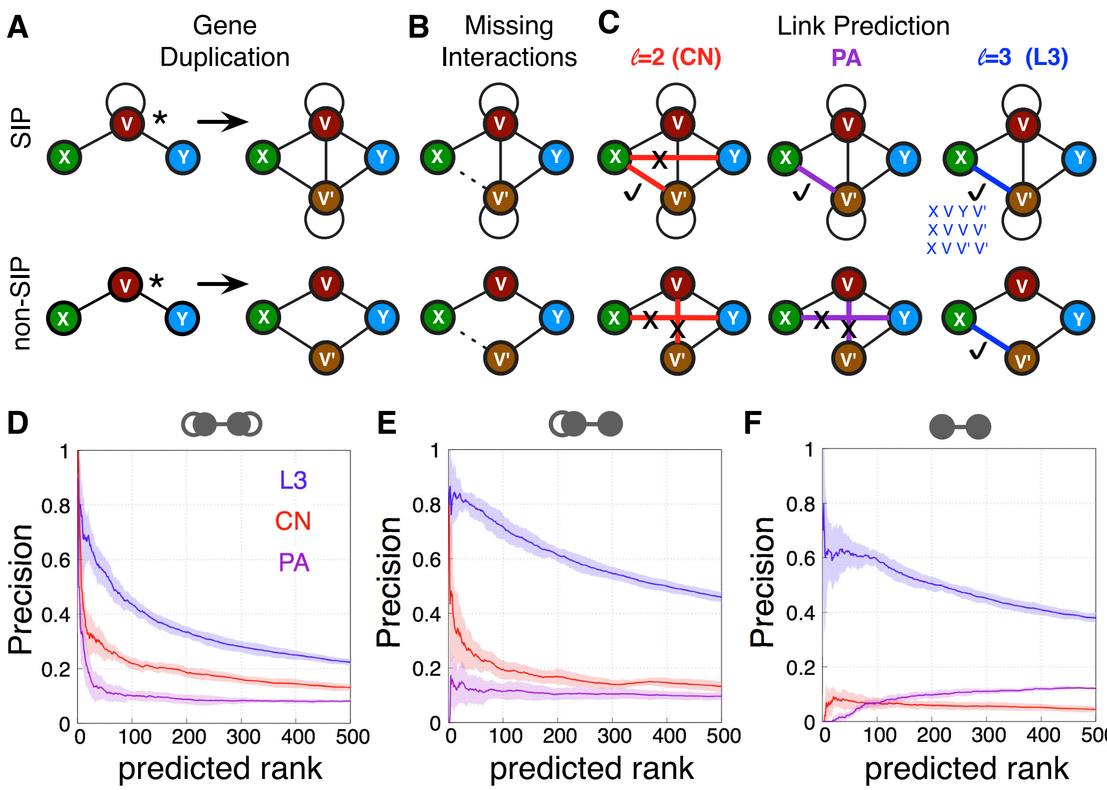

Supplementary Figure 2: **From gene duplication to link prediction.** (**A**) A key evolutionary mechanism responsible for the emergence of novel proteins is gene duplication [16, 17]. If protein V duplicates, the duplicated node (V') will (at least initially) retain the links of the original protein. As self-interacting proteins (SIPs) can interact with a copy of themselves, the new protein (V') will have a direct link to the original protein (V) as well. On the contrary, a duplicate of a nonSIP will lack a direct link to its paralog. Consequently, gene duplication leads to very different network patterns for SIPs and nonSIPs. (**B**) Finding a missing (dotted) link is a substantially different problem in the two cases. (**C**) The $\ell = 2$ TCP is represented by counting Common Neighbors (CN) and compared with the Preferential Attachment principle (PA) as well as with our $\ell = 3$ connectivity. In the neighborhood of a SIP, all 3 methods are able to identify the missing link, as indicated by the top predicted, colored links. The blue letters indicate the 3 distinct paths at $\ell = 3$ contributing to the top predicted link. TCP is prone to predict false positives even around SIPs. Around a nonSIP, TCP and PA fails completely, while the $\ell = 3$ connectivity principle is expected to succeed. (**D** to **F**) Computational cross-validation of Fig. 3B, regarding SIPs. We find that L3 (blue) outperforms CN and PA, even between pairs of SIPs in D), where TCP is expected to be valid. The shaded range around the curves indicates the standard deviation of the results over 10 realizations.

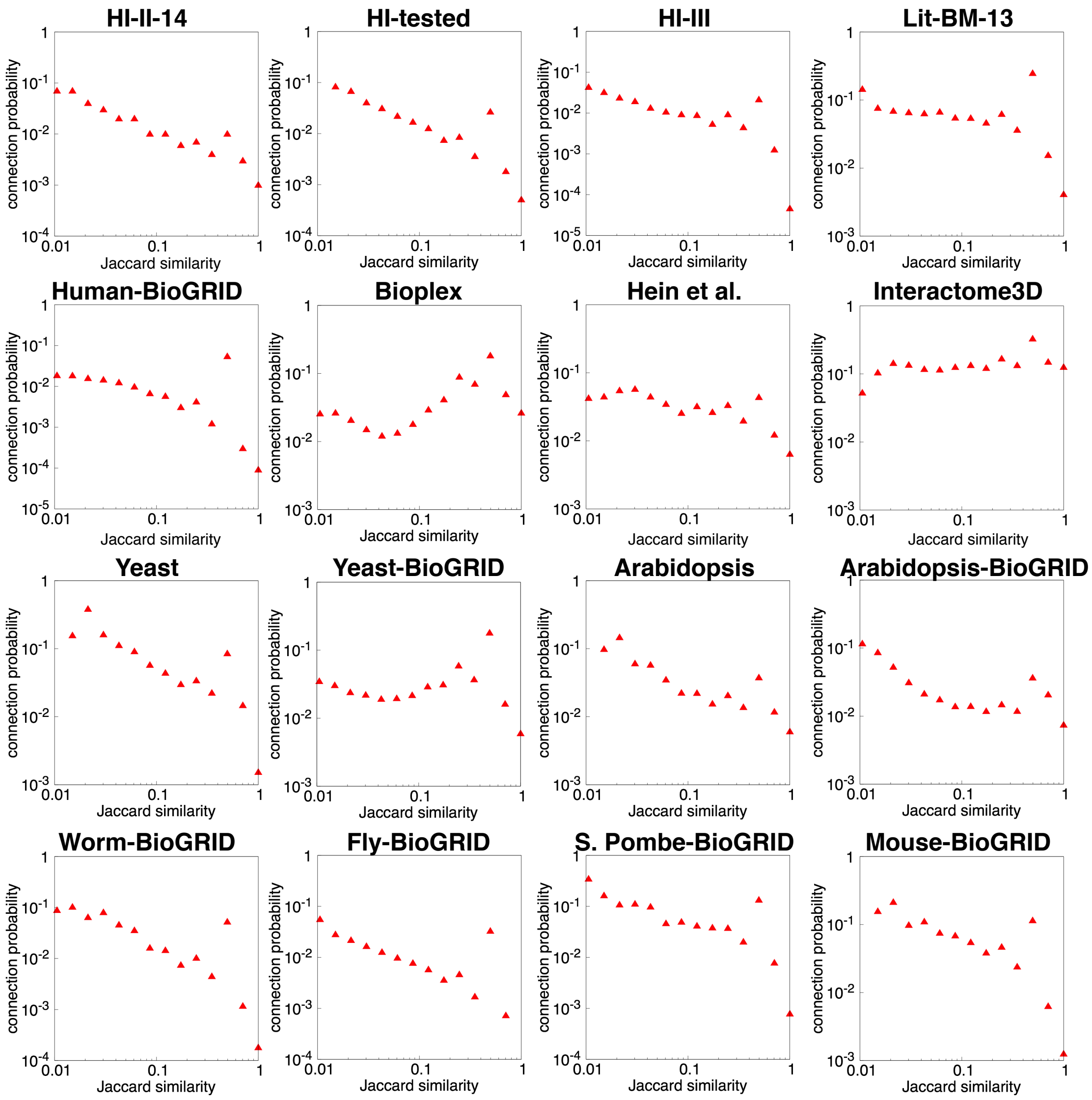

Supplementary Figure 3: **Connection probability vs Jaccard similarity.** Instead of a strong positive correlation expected by TCP, we typically observe a negative correlation between node similarity and connection probability as illustrated here for the network datasets listed in Supplementary Table 2. The protein pairs are logarithmically binned based on their Jaccard similarity values and the connection probability is measured in each bin.

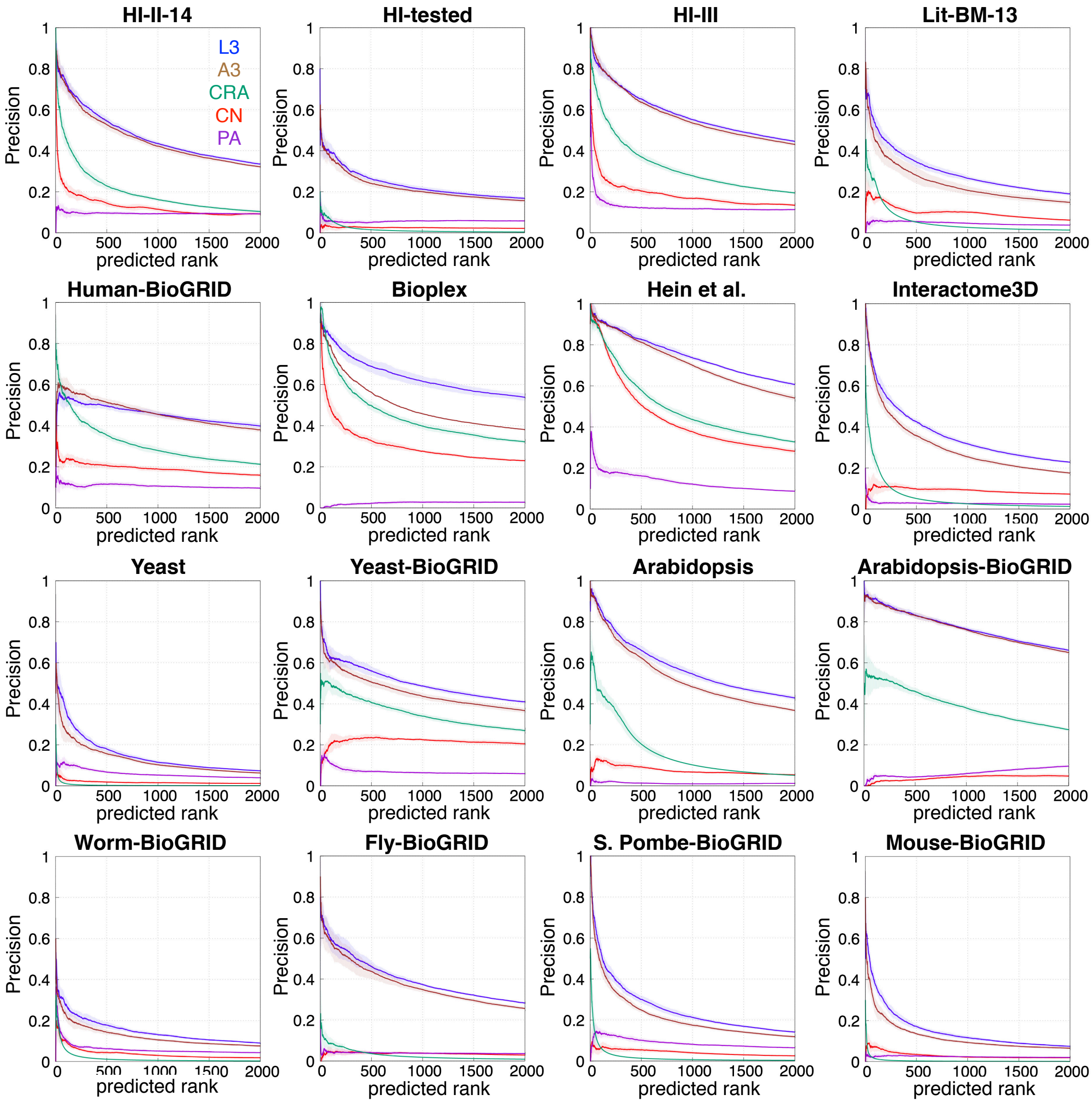

Supplementary Figure 4: **Computational cross-validation for all studied networks.** In the computational cross-validation 50% of the links are randomly selected as the set of input interactions, used for link prediction, tested against the other half of the data for the top 2000 predicted links, averaged over 10 trials. While the relative performance of literature methods (CRA, CN, PA) depends on the studied network, L3 outperforms all methods for all cases, as illustrated here for the network datasets listed in Supplementary Table 2. L3 (blue, in Eq. (1)) always performs at least as well as the unnormalized A^3 measure (brown). The shaded range around the curves indicates the standard deviation over 10 realizations.

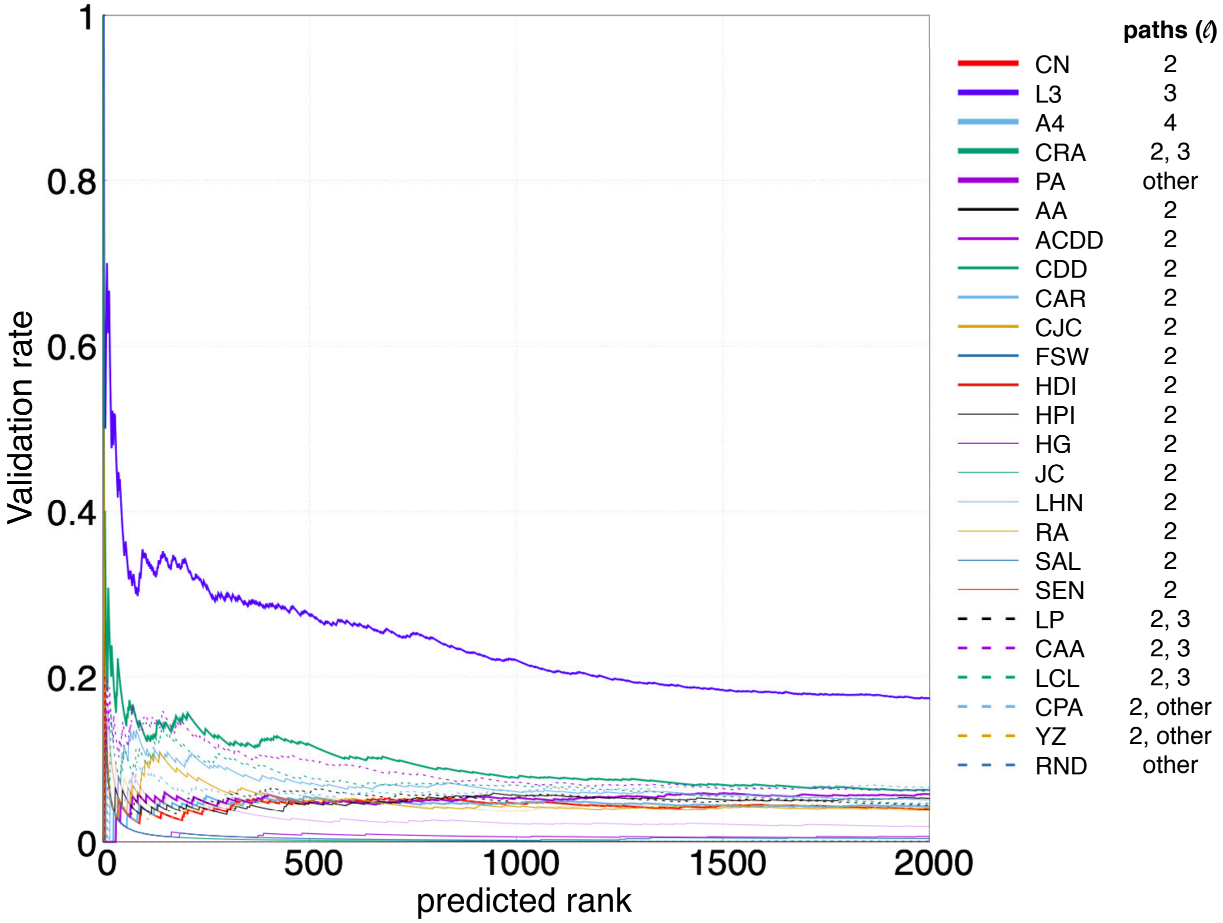

Supplementary Figure 5: **High-Throughput evaluation of various link prediction methods.** Here we test the predicted links based on HI-tested against HI-III [18] for link prediction methods listed in Supplementary Table 1, as well as for L3. Amongst the literature techniques, CRA shows the best initial performance, therefore being selected for the pairwise testing experiments in Fig 3g-h. CRA's relatively good performance is attributed to the fact that CRA is not an independent method from L3, but provides a subset of the links identified by L3.

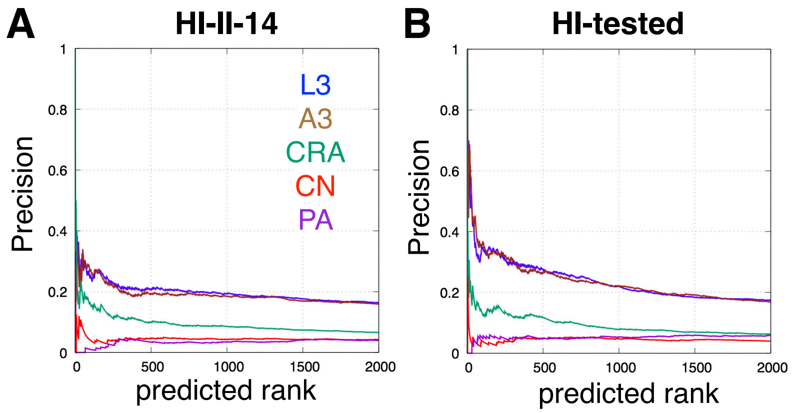

Supplementary Figure 6: **High-Throughput validation on HI-II-14 and HI-tested.** Here we test the predicted links against HI-III [18] to see the change in performance due to the filtering when selecting HI-tested from HI-II-14 [1]. Overall, the results look qualitatively very similar when HI-II-14 (panel **A**) or HI-tested (panel **B**) is used as an input dataset. This is remarkable, especially since HI-tested has only a fraction (∼ 68%) of the links from HI-II-14. As in the computational cross-validation on these networks in Supplementary Fig. 4, L3 or the unnormalized version (A3) yields very similar results.

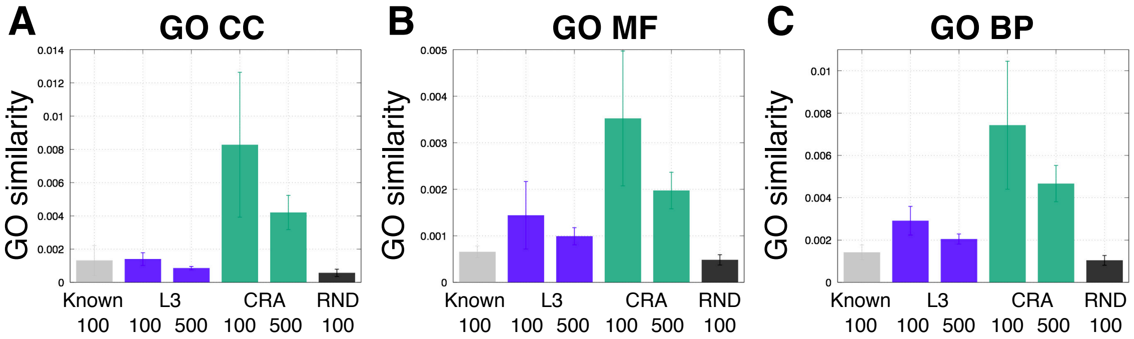

Supplementary Figure 7: **Functional similarity of the predicted links measured by GO-term similarity.** GO-term annotations [36, 37] are routinely applied to validate computationally predicted links in the literature [9, 38], instead of performing high-throughput validations or pairwise testing experiments. However, as we show here, proteins that are not connected but have a high topological similarity might be more similar in their GO-annotations [37] than interacting proteins. We observe the same qualitative behavior for each of the three categories, cellular component (panel **A**, "CC"), molecular function (panel **B**, "MF") and biological process (panel **C**, "BP"). While there is generally only a weak difference between the GO-term similarity of the known ("Known") interactions and random pairs ("RND"), the topologically similar pairs predicted by CRA show a high GO-term similarity, even though connected much less likely. Thus, our observations question the legitimacy of using GO-terms or functional annotations to evaluate the quality of the predicted physical interactions. GO-term similarity between two proteins [11] is quantified as $2/\min(n_i)$, where n_i is the total number of proteins annotated with shared term i between the two proteins. The error bars indicate the statistical error of the indicated average similarities.

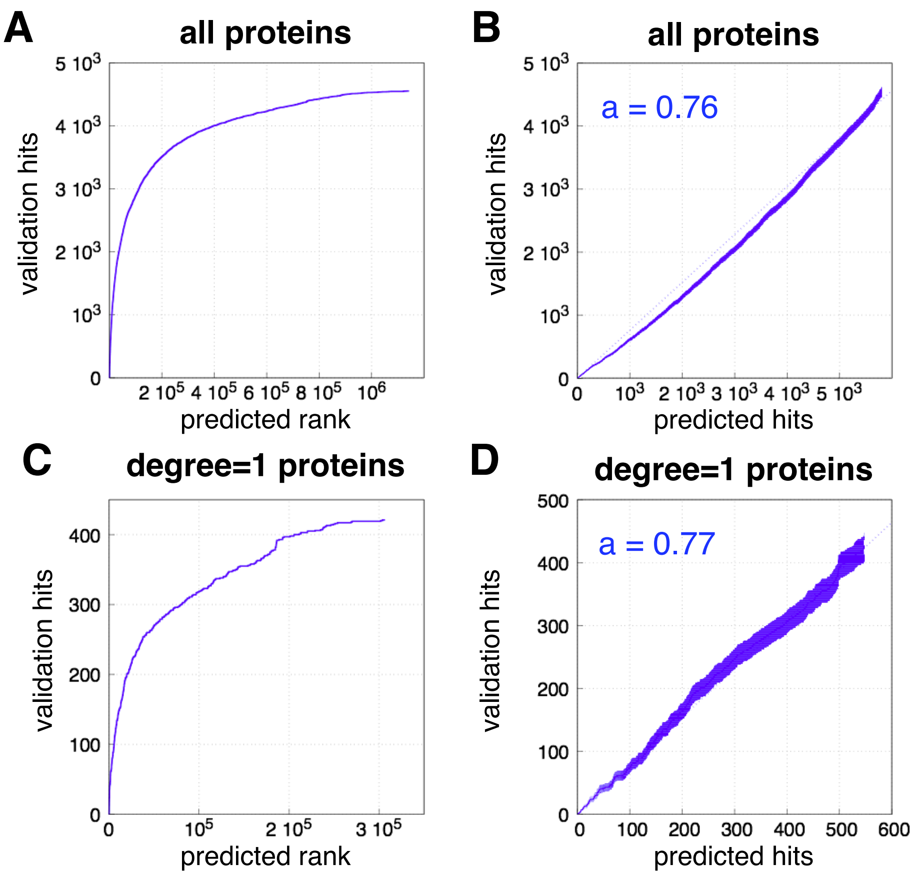

Supplementary Figure 8: **Assigning probabilities to the L3 predictions.** (**A**) When considering the full range of predictions, the L3 rank is found to be very informative on the validation probability of the predicted link, as indicated by the high-throughput validation results on HI-tested. (**B**) Based on the estimated validation probabilities for each pair, we can provide close estimates for the number of validated interactions for the full range of predicted ranks. (**C**)-(**D**) The same as A)-B), ranked and filtered for the worst-case scenario, where at least one of the nodes has degree equal to 1. We see a similar relationship as before in A)-B), with a consistent prefactor, indicating that L3 is similarly reliable even for low-degree proteins. The shaded area in C) and D) indicates the expected standard deviation.

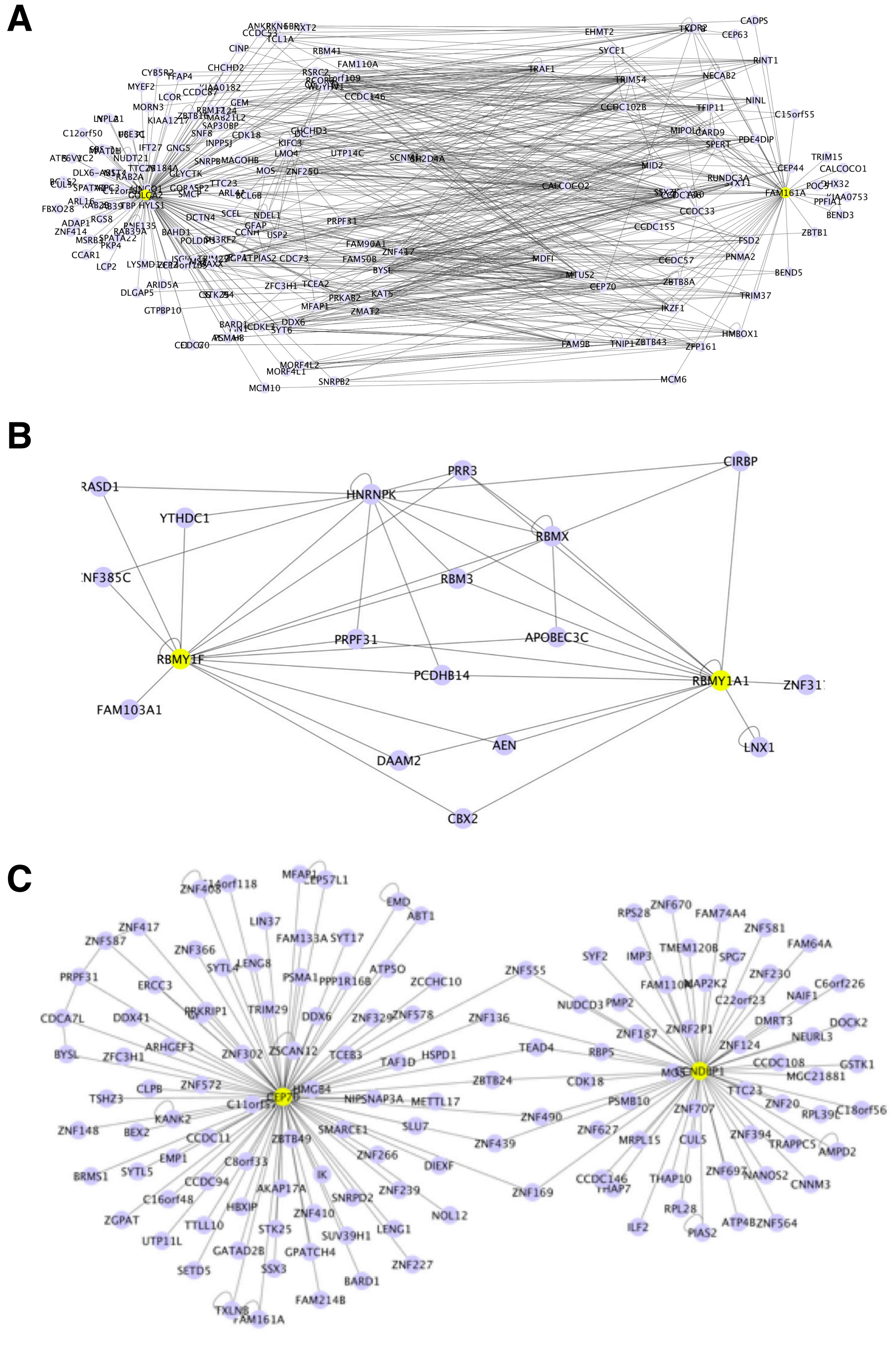

Supplementary Figure 9: **Link prediction examples.** (**A**) The top predicted L3 link on HI-tested between FAM161A and GOLGA2, validated by both HI-III and our pairwise testing experiment. Here we show all neighbors of these two proteins (yellow) and the connections between them. Interestingly, FAM161A and GOLGA2 have no neighbors in common, placing their interaction beyond the reach of TCP based methods. (**B**) The top predicted link of CRA is between two SIPs, characterized by high level of connectivity at both $\ell = 2$ and $\ell = 3$ paths, validated by both the HT and PT tests. (**C**) An example of two nodes sharing a number of common neighbors (7), without any $\ell = 3$ connectivity. Both nodes are nonSIPs and they are not connected in HI-III.

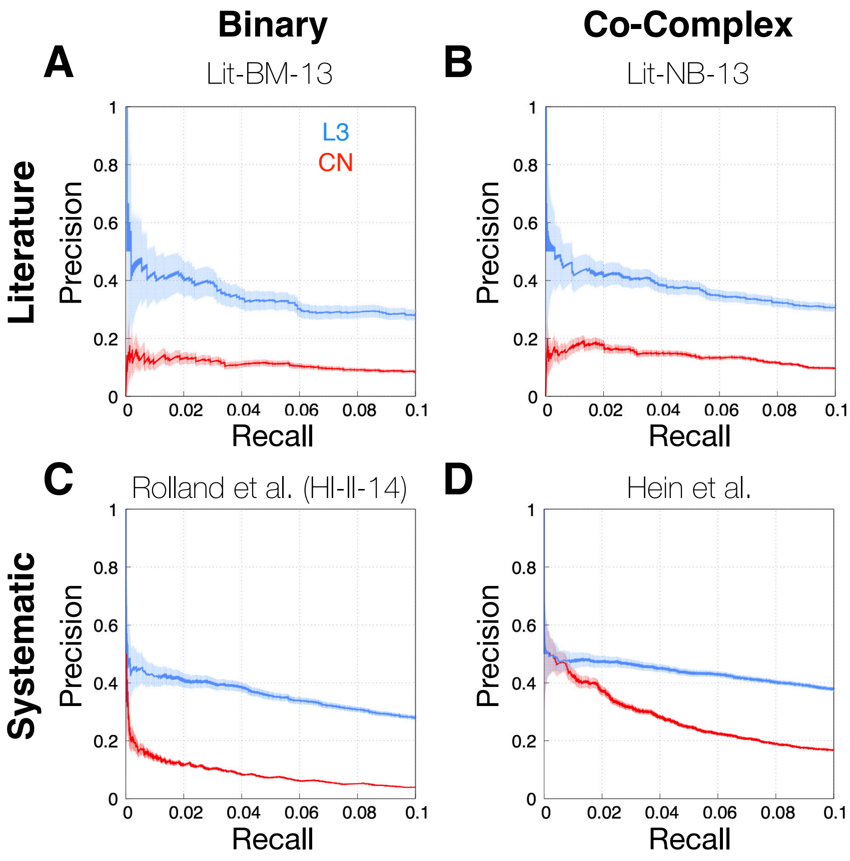

Supplementary Figure 10: **L3 outperforms Common Neighbors (CN) on PPI networks.** Leave-one-out cross-validation of CN and L3 on the four possible PPI data sources shown in Fig. 2. We perform an exhaustive leave-one-out cross-validation, with all links tested one-by-one on the four PPI data sources, arising from literature curation with multiple evidences (**A** and **B** [1]) or systematic screens (**C** [1] and **D** [14]). Precision is the fraction of known interacting proteins vs. all predicted pairs, while recall stands for the fraction of predicted PPIs compared to the total number of PPIs. As in Fig. 2, we find that L3 outperforms CN in all cases. Generally, we see that our results stay qualitatively very similar to Fig. 2, apart from a lower top prediction achieved (especially in panels C and D). This is beacuse not all positive predictions are detected, but only a fraction of them in the incomplete input data. The observed top precision is informative, serving as a lower bound for the completeness of the input network. The shaded area indicates the statistically expected standard deviation.

Supplementary References

[1] Rolland T, et al., A proteome-scale map of the human interactome network. Cell 159:5, 1212-1226 (2014).
[2] Mosca R, Col A, Aloy P, Interactome3D: adding structural details to protein networks. Nature Methods 10(1):47-53 (2013).
[3] Huttlin EL et al., Architecture of the human interactome defines protein communities and disease networks. Nature **545** 505-509 (2017).
[4] Clauset A., Moore C. & Newman M. E. J. Hierarchical structure and the prediction of missing links in networks. Nature 453, 98?101 (2008).
[5] Lü L & Zhou T, Link prediction in complex networks: A survey, Physica A 390 1150-1170 (2011).
[6] Wang, P., Xu, B. W., Wu, Y. R. & Zhou, X. Y., Link prediction in social networks: the-state-of-the-art. Sci. China Inform. Sci. 58, 1?38 (2015).
[7] Newman MEJ, Clustering and preferential attachment in growing networks. Phys Rev E 64, 025102 (2001).
[8] Barabási AL, Jeong H, Néda Z, Ravasz, E, Schubert A & Vicsek T, Evolution of social network of scientific collaboration. Physica A, 311(3-4):590-614 (2002).
[9] Cannistraci CV, Alanis-Lobato G & Ravasi T, From link-prediction in brain connectors and protein interactomes to the local-community-paradigm in complex networks Sci. Rep 3, 1613 (2013).
[10] Chua HN, Sung WK & Wong L, Exploiting indirect neighbours and topological weight to predict protein function from protein–protein interactions, Bioinformatics, 22:1623–1630 (2006).
[11] Menche J et al, Uncovering disease-disease relationships through the incomplete interactome. Science 347:6224, 1257601-1 (2015).
[12] Brun, C. et al. Functional classification of proteins for the prediction of cellular function from a protein-protein interaction network. Genome biology 5, R6 (2003).
[13] Liu, G.,Wong, L. & Chua, H. N. Complex discovery from weighted PPI networks. Bioinformatics 25, 1891?1897 (2009).
[14] Hein MY et al, A Human Interactome in Three Quantitative Dimensions Organized by Stoichiometries and Abundances Cell 163, 712-723 (2015).
[15] Ispolatov I, Yuryev A, Mazo I & Maslov S., Binding properties and evolution of SIPs in protein-protein interaction networks. Nucleic Acids Res. 2005 Jun 27;33(11):3629-35. (2005).
[16] Zhang J. Evolution by gene duplication: an update. Trends in Ecology & Evolution. 18 (6): 292?8. (2003).
[17] Dehal, P. & Boore, J. L. Two Rounds of Whole Genome Duplication in the Ancestral Vertebrate. PLoS Biol 3(10): e314. (2005).
[18] Preliminary CCSB human interactome, screens 1,2,3. `http://interactome.dfci.harvard.edu/index.php?page=login&lg=/H_sapiens/index.php?page=download_newrelease`
[19] Kunegis J, De Luca EW & Albayrak S. The Link Prediction Problem in Bipartite Networks. Computational Intelligence for Knowledge-Based Systems Design (Lecture Notes in Computer Science vol 6178) Berlin: Springer pp 380-389 (2010).
[20] Daminelli S, Thomas JM, Durán C & Cannistraci CV. Common neighbours and the local-community-paradigm for topological link prediction in bipartite networks. New J. Phys. **17** 113037 (2015).
[21] Stark C, Breitkreutz BJ, Reguly T, Boucher L, Breitkreutz A, Tyers M. Biogrid: A General Repository for Interaction Datasets. Nucleic Acids Res. Jan1; 34:D535-9 BioGRID Release 3.4.143 (???)
[22] Jaccard P, The distribution of flora in the alpine zone. The New Phytologist, 11, 37-50.
[23] Yu et al., High Quality Binary Protein Interaction Map of the Yeast Interactome Network. Science, 322(5898): 104-110 (2008).
[24] Arabidopsis Interactome Mapping Consortium, Evidence for Network Evolution in an Arabidopsis Interactome Map. Science 333, 6042 pp 601-607 (2011).
[25] Yang J & Zhang XD, Predicting missing links in complex networks based on common neighbors and distance. Sci. Rep. 6: 38208 (2016).
[26] Lü L, Jin CH & Zhou T, Similarity index based on local paths for link prediction of complex networks. Phys. Rev. E 80, 046122 (2009).
[27] Berman HM et al. The Protein Data Bank. Nucleic Acids Research, 28: 235-242. (2000).
[28] Dreze M, Monachello D, Lurin C, Cusick ME, Hill DE, Vidal M & Braun P. High-quality binary interactome mapping. Methods Enzymol. 470:281-315. (2010).
[29] `http://horfdb.dfci.harvard.edu/`
[30] Goldberg DS & Roth FP, Assessing experimentally derived interactions in a small world Proceedings of the National Academy of Sciences 100:4372–4376. (2003).
[31] Adamic LA & Adar E, Friends and Neighbors on the Web. Social Networks 25, 211-230 (2003).
[32] Zhou T, Lu LY & Zhang YC, Predicting missing links via local information. Eur. Phys. J. B 71, 623-630 (2009).
[33] Hanley JC & McNeil BJ, The meaning and use of the area under a receiver operating characteristic (ROC) curve. Radiology 143 29-36 (1982).
[34] Herlocker JL, Konstan JA, Terveen LG & Riedl JT, Evaluating collaborative filtering recommender systems. ACM T. Inform. Syst. 22, 5-53, (2004).
[35] Wang Y, Wang L, Li Y, He D, Chen W & Liu TY. A Theoretical Analysis of Normalized Discounted Cumulative Gain (NDCG) Ranking Measures. In Proceedings of the 26th Annual Conference on Learning Theory (COLT 2013).
[36] Ashburner M, et al., Gene ontology: tool for the unification of biology. Nature genetics 25:25-29. (2000).
[37] Himmelstein D et al. Gene-ontology: Initial zenodo release. Zenodo. (2015). `DOI: 10.5281/zenodo.21711` Version: Last updated on 2016-05-02 18:05:21.

[38] You, Z.-H., Lei, Y.-K., Gui, J., Huang, D.-S. & Zhou, X. Using manifold embedding for assessing and predicting protein interactions from high-throughput experimental data. Bioinformatics (Oxford, England) 26, 2744?2751 (2010).

[39] Goldberg DS & Roth FP, Assessing experimentally derived interactions in a small world Proceedings of the National Academy of Sciences 100:4372–4376. (2003).

[40] Kuchaiev et al., Geometric de-noising of protein-protein interaction networks. PLoS Comp Biol 5(8): e1000454 (2009).

[41] Alanis-Lobato G, Mier P, & Andrade-Navarro M, The latent geometry of the human protein interaction network. Bioinformatics, 1-9 (2018).

[42] Zhu B & Xia Y, An information-theoretic model for link prediction in complex networks. Scientific Reports, 5:13707 (2015).
