# Supplementary material for: Network-based prediction of protein interactions
Source: Nat Commun. 2019 Mar 18;10:1240. doi: 10.1038/s41467-019-09177-y (PMC6423278; doi:10.1038/s41467-019-09177-y)
Supplement: Supplementary file 3 — Reporting Summary [file 41467_2019_9177_MOESM3_ESM.pdf]

nature research

Corresponding author(s): Albert-Laszlo Barabasi

Last updated by author(s): Feb 9, 2019

Reporting Summary

Nature Research wishes to improve the reproducibility of the work that we publish. This form provides structure for consistency and transparency in reporting. For further information on Nature Research policies, see Authors & Referees and the Editorial Policy Checklist.

Statistics

For all statistical analyses, confirm that the following items are present in the figure legend, table legend, main text, or Methods section.

n/a	Confirmed	
☐	☒	The exact sample size (*n*) for each experimental group/condition, given as a discrete number and unit of measurement
☐	☒	A statement on whether measurements were taken from distinct samples or whether the same sample was measured repeatedly
☐	☒	The statistical test(s) used AND whether they are one- or two-sided<br>*Only common tests should be described solely by name; describe more complex techniques in the Methods section.*
☒	☐	A description of all covariates tested
☒	☐	A description of any assumptions or corrections, such as tests of normality and adjustment for multiple comparisons
☐	☒	A full description of the statistical parameters including central tendency (e.g. means) or other basic estimates (e.g. regression coefficient) AND variation (e.g. standard deviation) or associated estimates of uncertainty (e.g. confidence intervals)
☐	☒	For null hypothesis testing, the test statistic (e.g. *F*, *t*, *r*) with confidence intervals, effect sizes, degrees of freedom and *P* value noted<br>*Give P values as exact values whenever suitable.*
☒	☐	For Bayesian analysis, information on the choice of priors and Markov chain Monte Carlo settings
☒	☐	For hierarchical and complex designs, identification of the appropriate level for tests and full reporting of outcomes
☒	☐	Estimates of effect sizes (e.g. Cohen's *d*, Pearson's *r*), indicating how they were calculated

Our web collection on statistics for biologists contains articles on many of the points above.

Software and code

Policy information about availability of computer code

Data collection	No data collection software was used.
Data analysis	Figures, statistical tests and most data analyses have been generated and performed by publicly available softwares GnuPlot, R and Cytoscape. Predictions have been done by short custom codes, e.g. a straightforward implementation of Eq. (1) in C++.

For manuscripts utilizing custom algorithms or software that are central to the research but not yet described in published literature, software must be made available to editors/reviewers. We strongly encourage code deposition in a community repository (e.g. GitHub). See the Nature Research guidelines for submitting code & software for further information.

Data

Policy information about availability of data

All manuscripts must include a data availability statement. This statement should provide the following information, where applicable:

- Accession codes, unique identifiers, or web links for publicly available datasets
- A list of figures that have associated raw data
- A description of any restrictions on data availability

The human interactome, HI-III is available at Ref. [26]. Additional data that support the findings of this study are available within the supplementary information files or from the corresponding author upon reasonable request. The protein interactions from this publication have been submitted to the IMEx (http://www.imexconsortium.org) consortium through IntAct and assigned the identifier IM-26274.

Field-specific reporting

Please select the one below that is the best fit for your research. If you are not sure, read the appropriate sections before making your selection.

☒ Life sciences ☐ Behavioural & social sciences ☐ Ecological, evolutionary & environmental sciences

For a reference copy of the document with all sections, see nature.com/documents/nr-reporting-summary-flat.pdf

Life sciences study design

All studies must disclose on these points even when the disclosure is negative.

Sample size	The sample size for pairwise test was chosen based on preliminary leave one out cross-validation analyses to have sufficient power to detect a statistically significant difference between the tested methods.
Data exclusions	As a pre-established design criteria for the experiments, we excluded keratins from the pairwise testing analyses as described in Supplementary Note 1.
Replication	We applied a routine experimental protocol, that has been extensively tested in the past with excellent reproducibility. In our specific experiments, we included positive and negative benchmarks. One these positive benchmark sets tested reproducibility of previously detected interactions under the same conditions. For details see Supplementary Note 1.
Randomization	In addition to our computational predictions, we tested the predictions of one literature method (CRA), in addition to positive and negative benchmarks. For details see Supplementary Note 1.
Blinding	The predicted groups and benchmarks have been randomly shuffled and run through the experimental pipeline in a blind way.

Reporting for specific materials, systems and methods

We require information from authors about some types of materials, experimental systems and methods used in many studies. Here, indicate whether each material, system or method listed is relevant to your study. If you are not sure if a list item applies to your research, read the appropriate section before selecting a response.

Materials & experimental systems

n/a	Involved in the study	
☒	☐	Antibodies
☐	☒	Eukaryotic cell lines
☒	☐	Palaeontology
☒	☐	Animals and other organisms
☒	☐	Human research participants
☒	☐	Clinical data

Methods

n/a	Involved in the study	
☒	☐	ChIP-seq
☒	☐	Flow cytometry
☒	☐	MRI-based neuroimaging

Eukaryotic cell lines

Policy information about cell lines

Cell line source(s)	We used yeast strains Y8800 and Y8930 (MATa and MATa respectively), two strains derived from PJ69-4 James, P., Halladay, J., and Craig, E. A. (1996). Genomic libraries and a host strain designed for highly efficient two-hybrid selection in yeast. Genetics 144, 1425–1436) which harbor the following genotype: leu2-3,112 trp1-901 his3-200 ura3-52 gal4D gal80D GAL2-ADE2 LYS2::GAL1-HIS3 MET2::GAL7-lacZ cyh2R.
Authentication	Yeast strain verification was performed by growth on selective media to verify the genotype of the yeast strains.
Mycoplasma contamination	The cell lines were not tested for mycoplasma contamination.
Commonly misidentified lines (See ICLAC register)	No commonly misidentified cell lines were used.
